# Intravenous thrombolysis before endovascular treatment in acute posterior circulation occlusions, what's next?

**DOI:** 10.3389/fneur.2025.1631936

**Published:** 2025-07-16

**Authors:** Senta Frol, Matija Zupan, Panagiotis Papanagiotou, Pawel Kermer

**Affiliations:** ^1^Department of Vascular Neurology, University Medical Center Ljubljana, Ljubljana, Slovenia; ^2^Faculty of Medicine, University of Ljubljana, Ljubljana, Slovenia; ^3^Clinic for Diagnostic and Interventional Neuroradiology, Klinikum Bremen Mitte, Bremen, Germany; ^4^Department of Radiology, Aretaieion University Hospital, National and Kapodistrian University of Athens, Athens, Greece; ^5^Department of Neurology, Nordwest-Krankenhaus Sanderbusch, Friesland Kliniken GmbH, Sande, Germany; ^6^Department of Neurology, University Medicine Göttingen, Göttingen, Germany

**Keywords:** vertebrobasilar occlusions, bridging therapy, adjunctive antithrombotics, revascularization, intra-arterial thrombolytics

## Introduction

Intravenous thrombolysis (IVT) is recommended as first-line therapy for acute ischemic stroke (AIS) (1, 2) while endovascular treatment (EVT) is the standard of care for AIS caused by large vessel occlusion (LVO). The value of administering IVT prior to EVT in patients with LVO has been debated for years. Some studies showed no advantage of associating IVT to EVT (3, 4), while others failed to demonstrate superiority or non-inferiority of EVT alone vs. EVT combined with IV recombinant tissue plasminogen activator (rt-PA) (5, 6). Meta-analysis confirmed that all studies designed as non-inferiority studies remained underpowered to establish the superiority of combining IVT and EVT (7).

Strokes in the posterior circulation account for ~20% of all ischemic stroke cases. Current guidelines recommend IVT combined with EVT for eligible patients with acute anterior circulation LVO, whether presenting to an EVT-capable center or to a primary stroke center (8). In contrast, there is limited data available regarding the efficacy and safety of IVT administered prior to EVT for vertebrobasilar (VB) occlusions.

This opinion article explores the latest insights on bridging therapy in VB occlusions and raises the question of potential adjunctive antithrombotic treatments for this patient population—topics that warrant further in-depth investigation.

## Methodology

For our narrative literature review, we searched PubMed and Scopus to 01/05/2025 for clinical studies, meta-analysis and real-world data reporting bridging therapy in posterior circulation. In addition, we searched references of related letters, reviews and editorials to identify other potentially eligible studies. To be eligible for the present narrative review, the studies had to be published full-text articles in English language. The search query included the following keywords: “Basilar artery,” “Posterior circulation,” “Vertebrobasilar,” “Stroke,” “Brain ischemia,” “Intravenous thrombolysis,” “Tissue plasminogen activator,” “Endovascular treatment,” “Bridging therapy,” “Adjunctive Anticoagulants and antiplatelets.”

## Clinical studies and meta-analysis

The two key randomized controlled studies in this population, BAOCHE (9) and ATTENTION (10), showed the benefit of EVT in AIS due to basilar artery occlusion (BAO), regardless of IVT administration. However, they were not specifically designed to evaluate an added benefit of IVT combined with EVT in these patients. Indeed, both trials reported substantially low IVT usage in their EVT arms, with 14% in BAOCHE (9) and 31% in ATTENTION (10). Moreover, they did not specifically analyze the subgroup of patients receiving both IVT and EVT to determine whether IVT provided additional benefits when combined with EVT. However, a secondary analysis of the ATTENTION trial comparing outcomes in patients receiving direct EVT and those receiving IVT+EVT did not demonstrate any advantages in enhanced safety and efficacy outcomes compared with those treated with direct EVT (11).

Four registry studies (12–15) assessed effectiveness and safety of combined IVT and EVT treatment in BAO, including 1,519 patients in total, of whom 570 received combined treatment. None of the studies was able to demonstrate any significant differences in the rates of moderate or favorable functional outcomes at 90 days, symptomatic intracerebral hemorrhage (sICH), mortality, and successful reperfusion (all), nor was direct EVT found to be safer (14).

Similarly, two smaller retrospective institutional or multicenter studies found no significant differences in functional outcomes and safety between IVT+EVT and EVT alone in these patients (16, 17), whereas a third study did reveal better odds of favorable clinical outcomes in the bridging IVT prior to EVT treatment arm (18). Moreover, a meta-analysis of four retrospective cohorts involving 1,127 patients revealed improved clinical outcomes and reduced mortality in combined treatment (19). Similarly, a recent meta-analysis including data from two randomized trials and 10 cohort studies, showed improved excellent functional outcomes and a lower risk of mortality, without an increased risk of sICH, in patients treated with combined IVT and EVT (20).

A recently published large, prospective study (21) reported that bridging IVT prior to EVT for acute BAO was associated with increased odds of favorable functional outcomes and reduced 90-day mortality, without raising safety concerns compared to direct EVT. This represents a significant advancement, as previous studies have not consistently demonstrated such benefit.

## Implications and emerging questions from recent study outcomes

A question emerges from the findings of the study by Pop et al. (21): could the benefits of bridging therapy be even greater with broader adoption of tenecteplase (TNK) over rt-PA? TNK possesses several pharmacological advantages, including greater fibrin specificity, a longer plasma half-life, and increased resistance to plasminogen activator inhibitor-1, and is now a recommended treatment option in the updated ESO guidelines (22). Further, intra-arterial (IA) TNK administration shows promise. The ANGEL-TNK trial demonstrated improved outcomes in anterior circulation stroke when IA TNK was given after successful recanalization (23), suggesting that a similar approach might be beneficial in VB stroke, where timely reperfusion of brainstem structures is critical. Prospective studies are essential to validate IA TNK strategies and to optimize dosing to minimize hemorrhagic risks.

Although TNK appears to be very promising, as shown in ATTEST-2 trial with an increased reperfusion rate of 8% compared to 4% with rt-PA (24), the role of thrombolysis in AIS due to LVO has been progressively shifting from the main therapeutic strategy to an adjunctive treatment to EVT. In addition to far superior reperfusion rates of 60 to 80% in EVT, its utilization is associated with less limitations related to time window, low ASPECT score or even an ongoing treatment with anticoagulants.

There is a growing body of literature regarding different adjunctive antithrombotic and IA thrombolytic modalities in EVT due to LVO AIS.

### Anticoagulants and antiplatelets

Our literature search retrieved only one study with anticoagulant use in LVO AIS during EVT. In MR-CLEAN MED trial, periprocedural intravenous acetylsalicylic acid and unfractionated heparin during EVT in LVO AIS in the anterior circulation were both associated with an increased risk of sICH, without evidence for a beneficial effect on functional outcome (25). However, to the best of our knowledge, there is no equivalent data for BAO in the literature.

We found eight non-randomized studies addressing add-on antiplatelets during EVT or within 24 h after IVT or EVT in patients with BAO, of which six were observational registry-based studies (26–31), one non-randomized trial (32), and one study combined data from a prospective registry and an open label, single-arm trial (33). Seven studies (26–31) compared add-on tirofiban, whereas one study eptifibatide (33) to no add-on antiplatelets. Three studies included solely BAO or dominant vertebral artery occlusion patients (26, 28, 30), whereas the other five studies described a subgroup of BAO patients or secondary analysis from posterior circulation studies, with uncertain proportion of BAO patients (27, 29, 31–33).

Chen et al. (28) included 645 patients with BAO within 24 h of symptom onset treated with EVT, of whom 363 received add-on tirofiban intravenously (0.4 μg/kg/min for 30 min followed by 0.1 μg/kg/min for up to 24 h). Although the choice of tirofiban use was left at the discretion of the treating physician, it was recommended under conditions with an increased risk of re-occlusion or distal embolization, such as stenting, angioplasty, a high number of passes, or atherosclerotic etiology. Tirofiban significantly reduced the 90-day disability level, mortality and the frequency of any ICH and symptomatic ICH. However, the authors speculated that the higher mortality and sICH in patients not receiving tirofiban were due to higher frequency of previous anticoagulation, IVT (20.2 vs. 17.1%) and IAT (18.8 vs. 8.0%).

Sun et al. (26) included 105 patients with atherosclerotic BAO undergoing EVT within 24 h of symptom onset. The treatment groups received either tirofiban (0.3–0.4 mg within 6–8 min IA and 0.15 μg/kg/min IV for 24 h) followed by dual antiplatelet therapy or immediate dual antiplatelet therapy. In tirofiban group, 24.3% received IVT and 20.3% IAT, whereas the rates were 6.5 and 32.3% in the no-tirofiban group. Tirofiban was used based on the treating physician's decision in cases with emergency stenting or balloon angioplasty, local new thrombosis or vascular dissection, and severe atherosclerotic lesions with a high risk of re-occlusion. EVT + tirofiban + dual oral antiplatelet therapy resulted in higher recanalization rates compared to EVT + dual oral antiplatelet therapy. However, the risk for sICH, 90-day mortality, and functional outcomes did not differ between the groups.

Yang et al. (30) included 662 LVO-AIS patients undergoing EVT within 24 h of symptom onset, of whom 158 had posterior BAO or dominant vertebral artery occlusion. Add-on tirofiban (0.25–1 mg IA, followed by 0.1 μg/kg/min IV for 24 h) was considered for patients with emergency stenting or angioplasty, presumed endothelial damage, instant reocclusion, or severe *in situ* atherosclerosis with a high risk of early reocclusion (34.7%). The proportion of patients with posterior circulation occlusion who received bridging IVT was not clearly stated by the authors. No significant differences in safety outcomes on sICH, total ICH and distal embolization and efficacy outcomes on artery recanalization and 3-month functional independence were observed between the tirofiban and non-tirofiban group in the posterior circulation stroke patients. Interestingly, tirofiban was significantly correlated with 90-day mortality reduction for posterior circulation stroke patients only.

Pan et al. (27) included 130 patients with BAO or vertebral artery occlusion of whom 49.2% received tirofiban (0.25–1 mg IA, followed by 0.1–0.15 μg/kg/min IV for 16–24 h) at the discretion of the treating physician for patients with severe residual stenosis (≥50%) after EVT, rescue treatment with stenting or angioplasty, ≥3 passes, or severe atherosclerosis with a high risk of reocclusion. IVT was received by 25.0% in the tirofiban and 39.4% in the no-tirofiban group. No significant differences were observed in functional outcome, sICH and mortality between the two groups.

Kellert al. (29) included 162 patients with LVO AIS, of whom 34 had posterior circulation AIS. Of them, 20 received tirofiban (recommended if stenting was performed or endothelial injury was feared). The IVT rates were high (65.0% in tirofiban group and 78.5% in the no-tirofiban group). Tirofiban did not influence recanalization rates. Fatal ICH occurred more frequently in tirofiban-treated patients in the entire cohort and was an independent predictor of poor outcome.

Zhao et al. (31) compared patients undergoing EVT with second generation stentrievers who did (*n* = 37 with posterior circulation occlusions) or did not (*n* = 25 with posterior circulation occlusions) receive add-on tirofiban. Tirofiban dosing was 0.25–0.5 mg IA, followed by 0.2–0.25 mg/h for 12–24 h. Typical indications for tirofiban at the interventionists' discretion were emergency stenting or angioplasty, successful recanalization by three or more passes, and severe atherosclerosis lesions with high possibility of reocclusion. In the tirofiban group, 11% received IVT and 24% IAT, whereas the respective numbers were 4 and 19% in the no-tirofiban group. Whereas, there were no differences in sICH and early reocclusion between the groups, tirofiban group had a significantly lower mortality and better odds of long-term functional independence.

Wu et al. (32) included 218 patients with LVO AIS undergoing EVT, of whom 40 patients had posterior circulation occlusions. Contrary to other studies, tirofiban was administered only as IA boluses with doses depending on the bleeding risk (maximum dose 10 μ/kg). Even after adjusting for stroke type (posterior vs. anterior circulation occlusion), patients treated with tirofiban compared with those without tirofiban had significantly higher rate of sICH as well as fatal ICH. This study directly compared different doses of tirofiban and showed the dose-dependent effect of the drug on ICH.

Contrary to the aforementioned studies, Ma et al. (33) was the only study to investigate add-on eptifibatide in patients with LVO-AIS treated with EVT within 24 h of onset. The posterior circulation subgroup comprised 46/162 patients, of whom 50.0% received eptifibatide (135–180 μg/kg in 5 min IV/IA, followed by 0.75–2 μg/kg/min IV for 24 h). Compared with controls, the eptifibatide group had significantly higher rates of successful recanalization and 90-day good functional outcomes, defined as mRS 0–2.

The recent ESO–ESMINT guidelines on acute management of BAO AIS suggest add-on antiplatelet treatment during or within 24 h after complicated EVT (defined as failed, imminent reocclusion, or need for additional stenting or angioplasty) and no concomitant IVT (34). The guidelines highlight that add-on antiplatelets should be regarded only as a rescue strategy after assessing the bleeding risk.

### Intra-arterial thrombolytics

In the POST-UK study, adjunct IA urokinase after near complete to complete reperfusion by EVT in LVO AIS, did not significantly increase the likelihood of survival without disability at 90 days (35).

In the CHOICE and PEARL trials, the use of adjunctive IA rt-PA on top of EVT resulted in higher likelihood of excellent 90-day neurological outcome compared to placebo or the medical management group, respectively (36, 37). Moreover, there were no significant differences in the rate of sICH or 90-day mortality between the two groups (37).

Adjunctive IA TNK to EVT in LVO AIS has been studied in three trials (POST-TNK, ATTENTION-IA, and ANGEL-TNK). In POST-TNK trial, adjunctive IA TNK did not significantly increase the likelihood of freedom from disability at 90 days in patients with LVO AIS presenting within 24 h of time last known well, who had achieved near complete to complete reperfusion after EVT (38). In patients with AIS due to acute posterior large or proximal vessel occlusion, IA TNK administered after successful recanalization was not associated with a statistically significant reduction in combined disability and mortality at 90 days (39). On the other hand, the ANGEL-TNK trial demonstrated that patients with anterior LVO AIS presenting 4.5 to 24 h from symptom onset may benefit from IA TNK following successful recanalization without increased bleeding or mortality risks (23).

While current data on IA TNK are largely focused on anterior circulation stroke, its use in the VB territory warrants further exploration. Given the complex angioarchitecture and risk of incomplete reperfusion in this region, targeted IA delivery could enhance thrombus resolution and minimize systemic exposure. Dedicated studies are needed to establish optimal dosing, timing, and patient selection for IA TNK in posterior circulation interventions.

In our opinion, in the light of the present data and its known pharmacologic characteristics, TNK seems to be the most promising adjunct agent in LVO AIS treated with EVT, which merits further investigation.

In the most recent registry analysis, authors reported 43.6% of patients in the IVT plus EVT group and 39.2% in the EVT-alone group had cardioembolic strokes (21). It would be informative to analyze outcomes within the IVT + EVT group among patients on direct oral anticoagulants (DOACs), particularly to determine whether IVT conferred any additional benefit or harm in this subgroup. DOACs are widely used, and current evidence indicates no significant safety concerns regarding IVT administration in DOAC-treated patients (40). Moreover, recent studies even suggest enhanced efficacy of IVT in this population (40, 41). Evaluating the safety and efficacy outcomes of DOAC-treated patients receiving IVT plus EVT compared to EVT alone would provide valuable insights. Additionally, the impact of TNK use in this setting remains an important emerging question; given TNK's enhanced fibrin specificity, treatment outcomes might be even more favorable.

## Conclusions and future perspectives

In conclusion, Pop et al. (21) presented the first compelling evidence supporting the benefits of IVT prior to EVT in the VB circulation. As TNK continues to be integrated into clinical practice, we believe that further prospective randomized trials, along with real-world data, are crucial to validate these findings. Additionally, it is important to assess the benefit of both intra-venous and IA TNK administration, as well as other adjunctive antithrombotic therapies used in conjunction with EVT. Notably, further investigation is needed on revascularization strategies in patients treated with DOACs.
